# Supplementary material for: The rediscovered motor-related area 55b emerges as a core hub of music perception
Source: Commun Biol. 2022 Oct 18;5:1104. doi: 10.1038/s42003-022-04009-0 (PMC9579133; doi:10.1038/s42003-022-04009-0)
Supplement: Supplementary file 2 — Supplementary Information [file 42003_2022_4009_MOESM2_ESM.pdf]

Supplementary Material for

The Rediscovered Motor-related Area 55b Emerges as a Core Hub of Music Perception

Tali Siman-Tov[1,2,*], Carlos R. Gordon[2,3], Netanell Avisdris[1,4,#], Ofir Shany[1,#], Avigail Lerner[1,3], Omer Shuster[5], Talma Hendler[1,2,3,6,†], Roni Y. Granot[5,†]

[1] Sagol Brain Institute Tel Aviv, Wohl Institute for Advanced Imaging, Tel Aviv Sourasky Medical Center, Tel Aviv, Israel
[2] Sackler School of Medicine, Tel Aviv University, Tel Aviv, Israel
[3] Sagol school of Neuroscience, Tel Aviv University, Tel Aviv, Israel
[4] School of Computer Science and Engineering, The Hebrew University of Jerusalem, Jerusalem, Israel
[5] Musicology Department, The Hebrew University of Jerusalem, Jerusalem, Israel
[6] School of Psychological Sciences, Tel Aviv University, Tel Aviv, Israel
[#] These authors contributed equally: Netanell Avisdris, Ofir Shany
[†] These authors jointly supervised this work: Talma Hendler, Roni Y. Granot

This PDF file includes:
Supplementary Tables 1-2
Supplementary Figures 1-7

Other Supplementary Materials for this manuscript include the following:
Supplementary Data 1-2

Supplementary Table 1. Pleasure ratings of musical excerpts by experimental condition.

Paradigm	Descriptive Statistics					Linear Mixed Model			
	Condition	Mean	SD	95% CI for Mean		numDF	denDF	F-value	p-value
				Lower	Upper				
Rhythm (n=62)	I	3.57	1.01	3.45	3.70	3	927	108.35	< 0.0001
	II	3.65	1.07	3.51	3.78				
	III	3.85	1.00	3.73	3.98				
	IV	2.67	1.02	2.55	2.80				
Melody (n=62)	I	1.80	0.96	1.68	1.92	3	927	321.87	< 0.0001
	II	3.90	1.01	3.78	4.03				
	III	3.37	0.99	3.25	3.50				
	IV	2.62	0.99	2.50	2.74				
Harmony (n=62)	T	3.49	1.15	3.35	3.64	3	927	61.71	< 0.0001
	VI	3.39	1.11	3.25	3.53				
	N	2.56	1.01	2.43	2.69				
	S	3.36	1.20	3.21	3.51				

Note. Descriptive statistics of pleasure ratings of musical excerpts across blocks of the same experimental condition (complexity level) for each musical paradigm (for boxplots, see Supplementary Figure 4). Linear mixed model showed significant effect of condition type on pleasure ratings in all three paradigms. I-IV, complexity levels 1-4, T, Tonic (regular cadence), VI, sixth degree (less regular cadence), N, Neapolitan (irregular cadence), S, scrambled version of the chord progression ending on the tonic, numDF, numerator degrees of freedom, denDF, denominator degrees of freedom.

Supplementary Table 2. Multiple linear regression results, repeated analysis with sphere-based region-of-interest.

Rhythm Paradigm (n=56)							
	Multiple Regression			Partial Regression Coefficients			
	R^2	F-value	p-value	Contrast Estimate	Age	Gender	MusEdu
AA	0.215	3.490	0.014*	-13.926***	-0.510	-4.830	-1.039
LRV-logit	0.239	4.005	0.007**	0.428***	-0.010	0.346	0.040
ENT	0.243	4.087	0.006**	0.039***	-0.000	0.028	0.004

Note. Dependent variables: Absolute Asynchrony (AA), Length of Resultant Vector - Logit (LRV-logit), Entropy of Relative-Phase Distribution (ENT). Independent variables: age, gender, musical education (MusEdu) and mean contrast estimate for the third condition of the Rhythm paradigm (vs. baseline) within a 6 mm sphere centered on the extra-auditory peak cerebral activation (MNI coordinates: 52, -2, 52).
*p < 0.05, **p < 0.01, ***p < 0.001

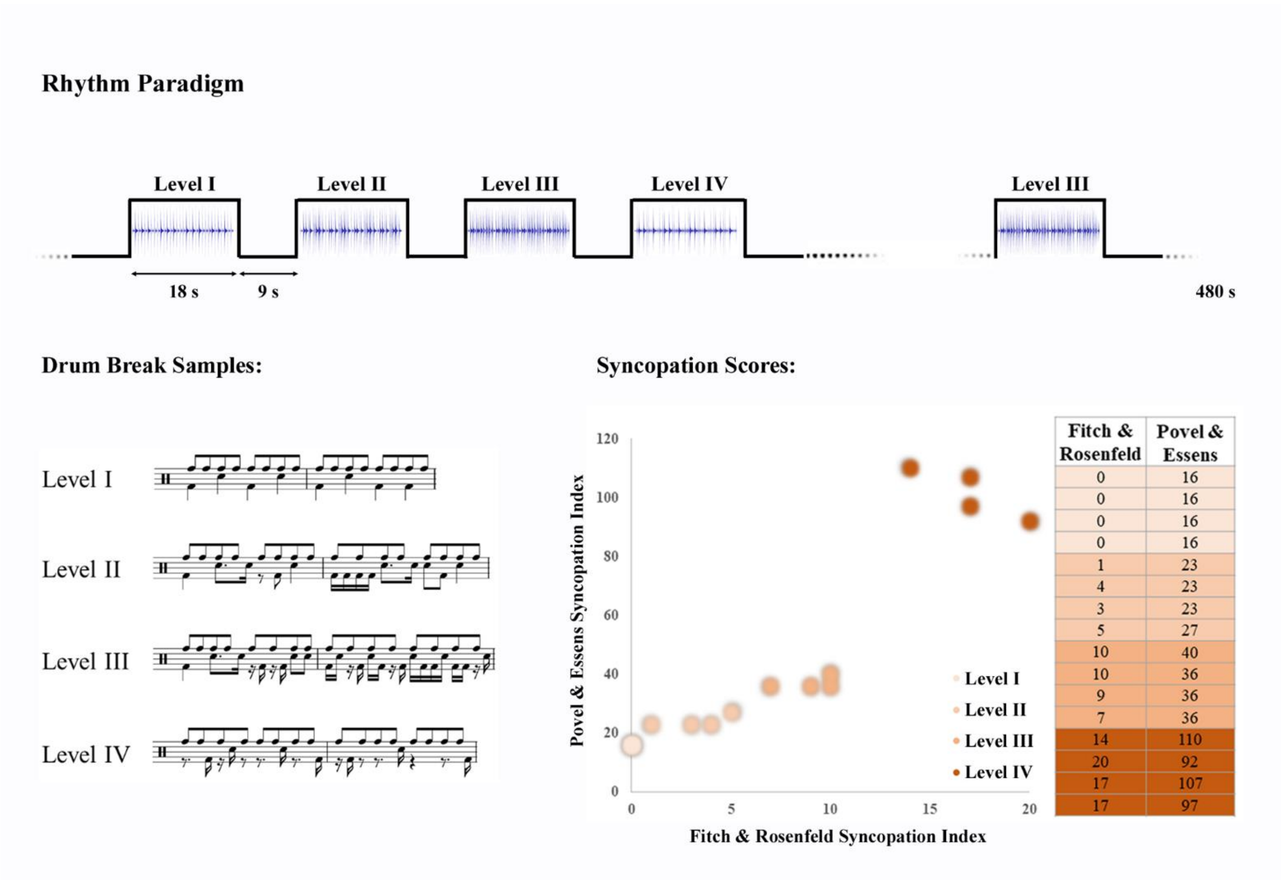

Fitch & Rosenfeld	Povel & Essens
0	16
0	16
0	16
0	16
1	23
4	23
3	23
5	27
10	40
10	36
9	36
7	36
14	110
20	92
17	107
17	97

Supplementary Figure 1. Schematic illustration of the Rhythm paradigm. The paradigm was composed of 16 blocks of drum breaks[95] at four levels of complexity. Rhythmic complexity was determined by two syncopation indices, proposed by Povel & Essens[96] and Fitch & Rosenfeld[97]. Syncopation scores are provided in the graph and table along with scores of example excerpts.

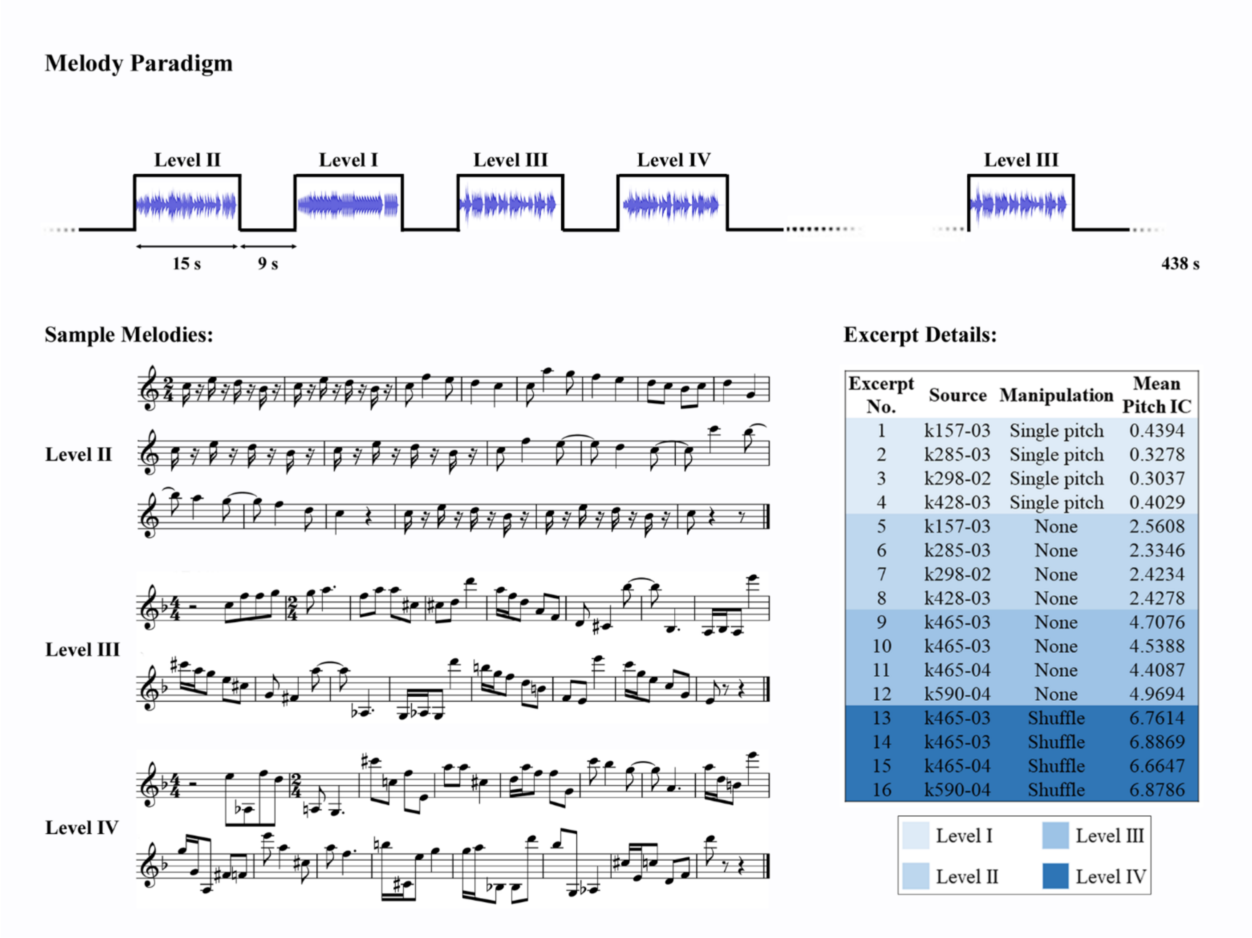

Excerpt No.	Source	Manipulation	Mean Pitch IC
1	k157-03	Single pitch	0.4394
2	k285-03	Single pitch	0.3278
3	k298-02	Single pitch	0.3037
4	k428-03	Single pitch	0.4029
5	k157-03	None	2.5608
6	k285-03	None	2.3346
7	k298-02	None	2.4234
8	k428-03	None	2.4278
9	k465-03	None	4.7076
10	k465-03	None	4.5388
11	k465-04	None	4.4087
12	k590-04	None	4.9694
13	k465-03	Shuffle	6.7614
14	k465-03	Shuffle	6.8869
15	k465-04	Shuffle	6.6647
16	k590-04	Shuffle	6.8786

Supplementary Figure 2. Schematic illustration of the Melody paradigm. The paradigm was composed of 16 blocks of instrumental melodies at four levels of complexity. The melodic excerpts were drawn from Mozart's string quartets. Melodic complexity was determined by averaging the pitch information content (IC) of each note in a tune as derived from the IDyOM algorithm[98,99]. The source of each excerpt (Kochel (K) and movement number), its mean pitch IC value and the manipulation performed are provided along with scores of example excerpts.

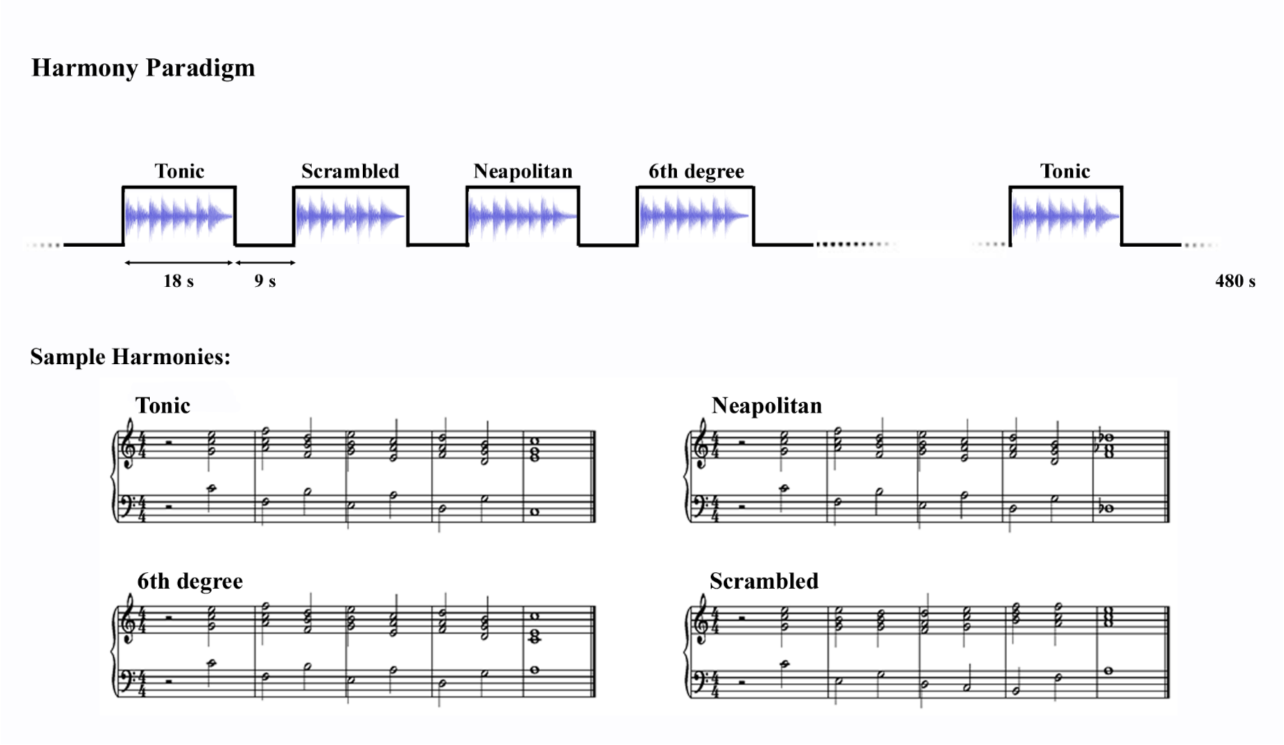

Supplementary Figure 3. Schematic illustration of the Harmony paradigm. The paradigm was composed of 16 blocks of harmonic progressions. Each block comprised three similar descending fifths progressions in different major keys. The progressions were ended on either the tonic chord (regular cadence), the sixth degree chord (less regular cadence) or the Neapolitan chord (irregular cadence). A scrambled version of the chord progression ending on the tonic was also included. Scores of chord progressions in C major are provided.

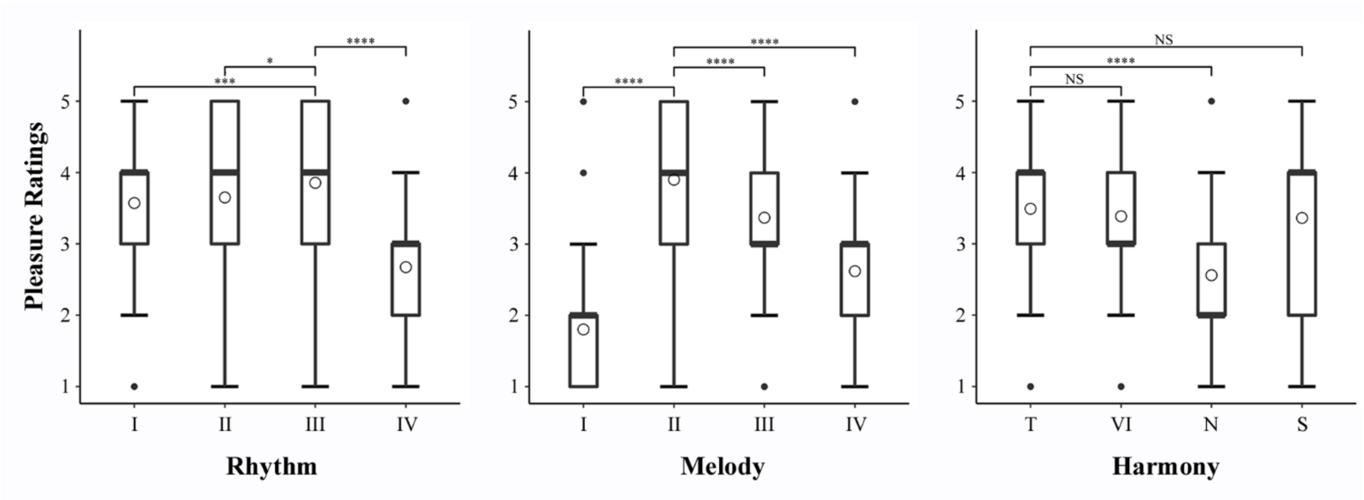

Supplementary Figure 4. Pleasure ratings of musical excerpts by experimental condition (complexity level). Boxplots comparing pleasure ratings (n=62) across blocks of each condition for each musical paradigm. Highest pleasure ratings were reported for the third, second and first conditions of the Rhythm, Melody and Harmony paradigms, respectively. For all boxplots, the horizontal line represents the median, the circle represents the mean, box limits represent the upper and lower quartiles, whiskers extend to 1.5 times the interquartile range and the black dots represent outliers. Asterisks represent significance level of post-hoc comparisons between conditions using Tukey's correction for multiple comparisons. I-IV, complexity levels 1-4, T, Tonic (regular cadence), VI, sixth degree (less regular cadence), N, Neapolitan (irregular cadence), S, scrambled version of the chord progression ending on the tonic. NS, non significant, *$p < 0.05$, **$p < 0.01$, ***$p < 0.001$, ****$p < 0.0001$. For further details, see Supplementary Table 1.

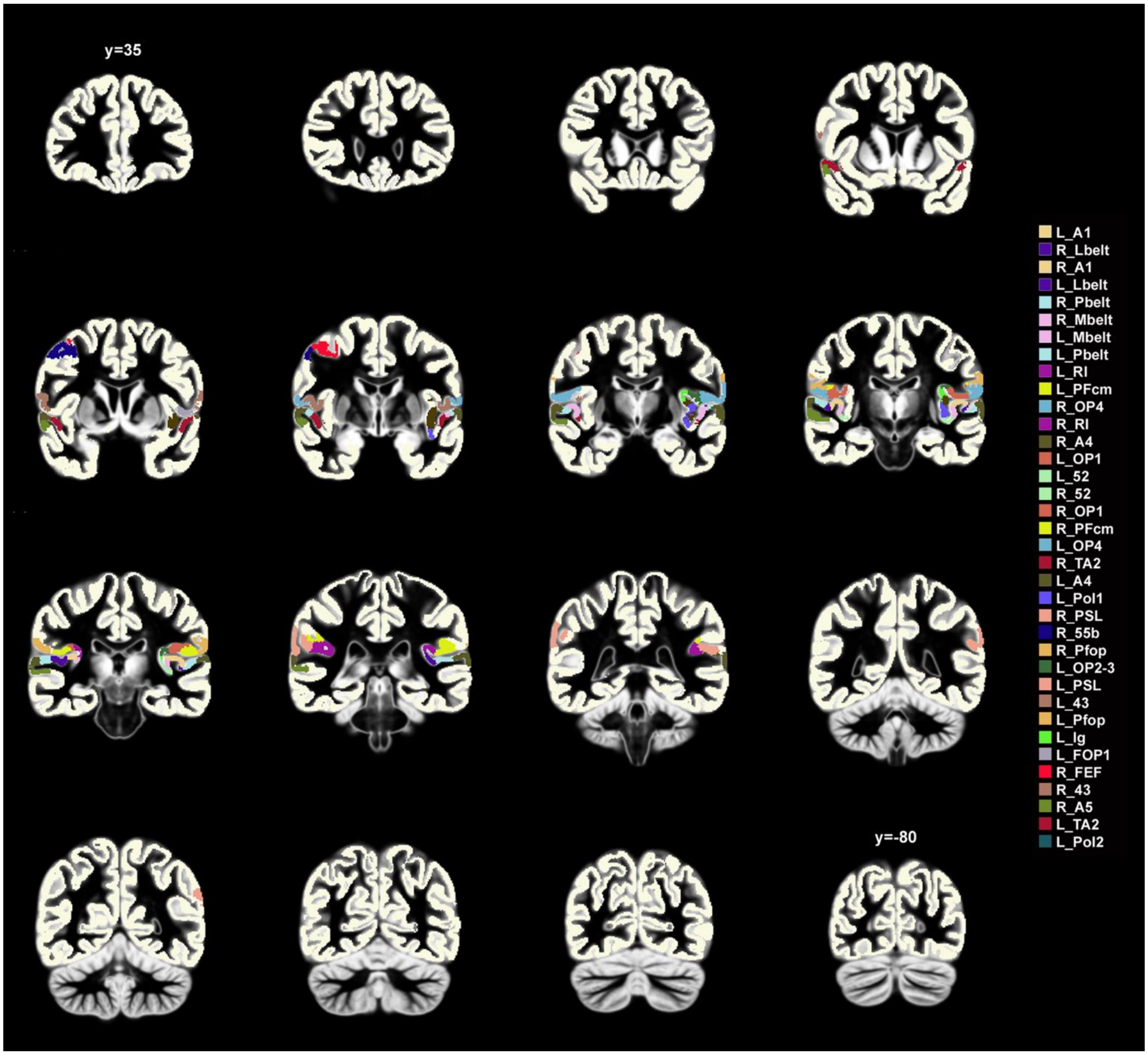

Supplementary Figure 5. HCP-MMP1's most activated parcels during passive listening to rhythmic phrases (Rhythm paradigm).

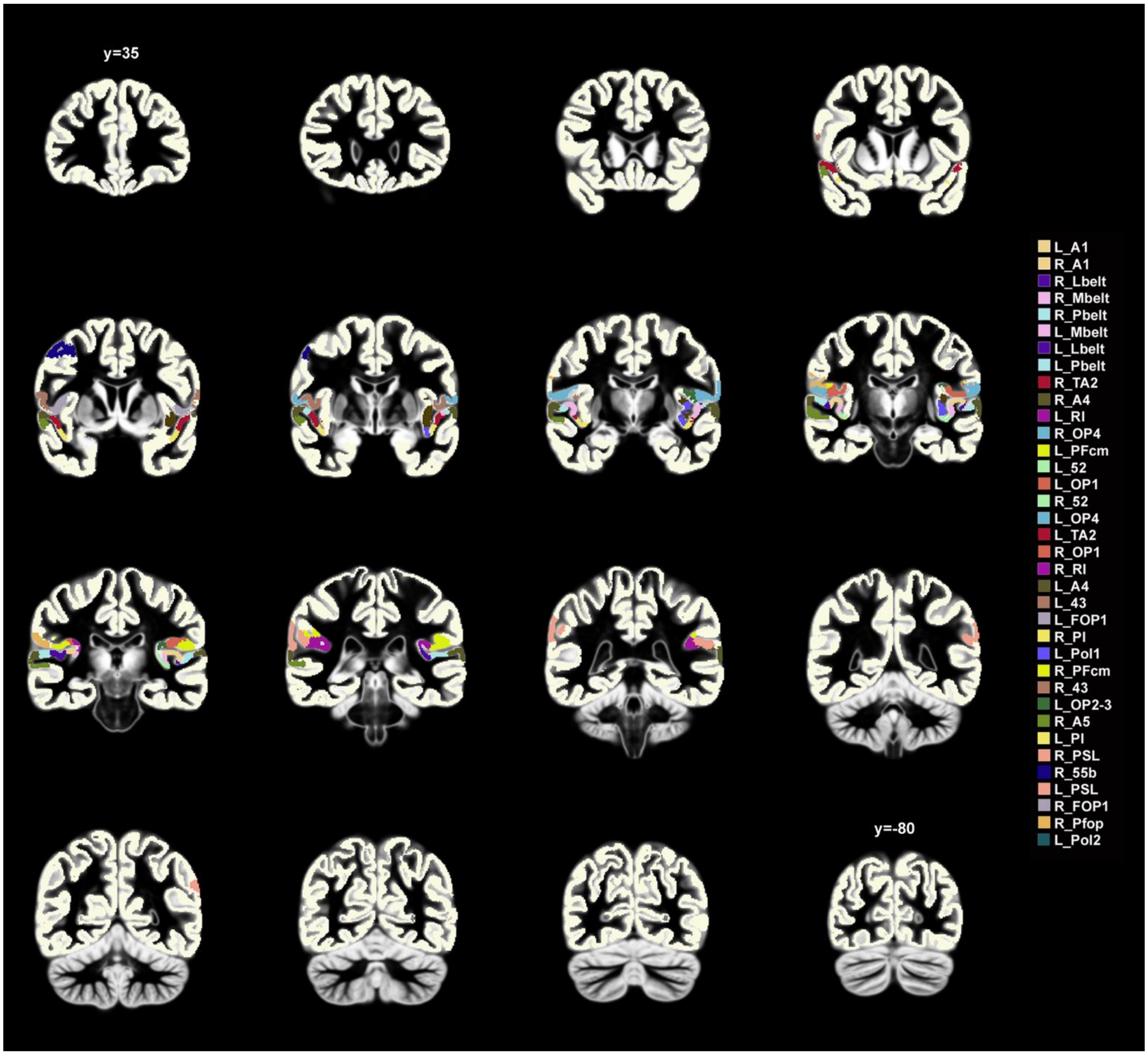

Supplementary Figure 6. HCP-MMP1's most activated parcels during passive listening to instrumental melodies (Melody paradigm).

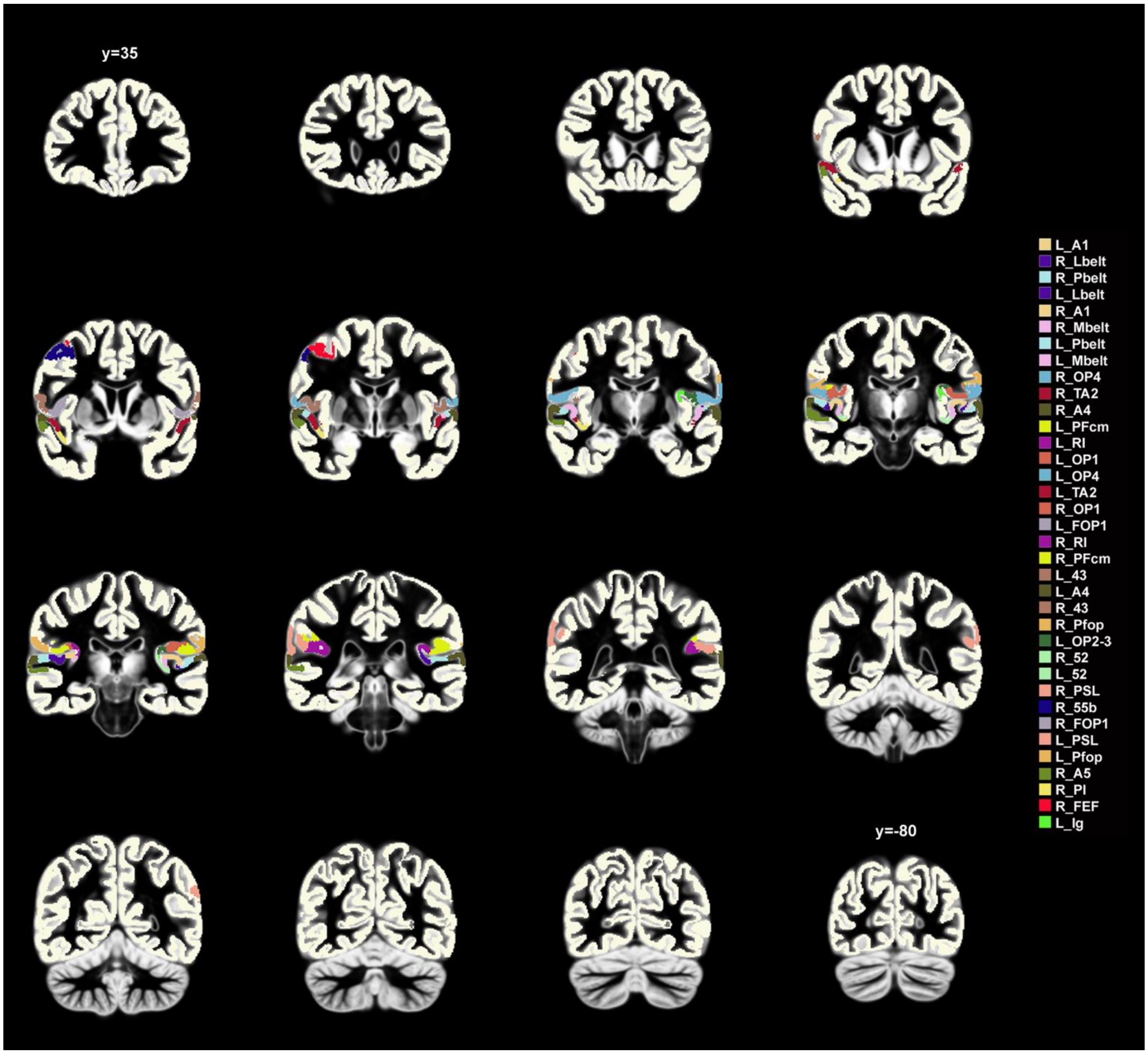

Supplementary Figure 7. HCP-MMP1's most activated parcels during passive listening to harmonic progressions (Harmony paradigm).
