# Supplementary material for: The rediscovered motor-related area 55b emerges as a core hub of music perception
Source: Commun Biol. 2022 Oct 18;5:1104. doi: 10.1038/s42003-022-04009-0 (PMC9579133; doi:10.1038/s42003-022-04009-0)
Supplement: Supplementary file 3 — Description of Additional Supplementary Files [file 42003_2022_4009_MOESM3_ESM.pdf]

Description of Additional Supplementary Files

File name: Supplementary Data 1

Description: Imaging studies included in the NeuroSynth meta-analysis of the term music

File name: Supplementary Data 2

Description: HCP-MMP1 - List of Regions
