# Supplementary material for: The rediscovered motor-related area 55b emerges as a core hub of music perception
Source: Commun Biol. 2022 Oct 18;5:1104. doi: 10.1038/s42003-022-04009-0 (PMC9579133; doi:10.1038/s42003-022-04009-0)
Supplement: Supplementary file 6 — Reporting summary [file 42003_2022_4009_MOESM6_ESM.pdf]

nature portfolio

Corresponding author(s): Tali Siman-Tov

Last updated by author(s): Aug 16, 2022

Reporting Summary

Nature Portfolio wishes to improve the reproducibility of the work that we publish. This form provides structure for consistency and transparency in reporting. For further information on Nature Portfolio policies, see our Editorial Policies and the Editorial Policy Checklist.

Statistics

For all statistical analyses, confirm that the following items are present in the figure legend, table legend, main text, or Methods section.

n/a	Confirmed	
☐	☒	The exact sample size (*n*) for each experimental group/condition, given as a discrete number and unit of measurement
☐	☒	A statement on whether measurements were taken from distinct samples or whether the same sample was measured repeatedly
☐	☒	The statistical test(s) used AND whether they are one- or two-sided<br>*Only common tests should be described solely by name; describe more complex techniques in the Methods section.*
☐	☒	A description of all covariates tested
☐	☒	A description of any assumptions or corrections, such as tests of normality and adjustment for multiple comparisons
☐	☒	A full description of the statistical parameters including central tendency (e.g. means) or other basic estimates (e.g. regression coefficient) AND variation (e.g. standard deviation) or associated estimates of uncertainty (e.g. confidence intervals)
☐	☒	For null hypothesis testing, the test statistic (e.g. *F*, *t*, *r*) with confidence intervals, effect sizes, degrees of freedom and *P* value noted<br>*Give P values as exact values whenever suitable.*
☒	☐	For Bayesian analysis, information on the choice of priors and Markov chain Monte Carlo settings
☒	☐	For hierarchical and complex designs, identification of the appropriate level for tests and full reporting of outcomes
☐	☒	Estimates of effect sizes (e.g. Cohen's *d*, Pearson's *r*), indicating how they were calculated

Our web collection on statistics for biologists contains articles on many of the points above.

Software and code

Policy information about availability of computer code

Data collection	Musical stimuli were generated using the Information Dynamics of Music (IDyOM) model (http://mtpearce.github.io/idyom/), KernScores dataset (http://humdrum.ccarh.org/), Sibelius 7.5 and Cubase Pro 9.5. Behavioral and imaging paradigms were presented using Presentation NBS 20.1. Behavioral data collection (finger tapping) was carried out using the TapArduino device (https://link.springer.com/article/10.3758/s13428-015-0671-3). Brain imaging was performed on a single 3T Siemens MAGNETOM Prisma scanner. The automated toolset Neurosynth (https://neurosynth.org/) was used to derive meta-analytic fMRI data.
Data analysis	Brain imaging data were analyzed using fMRIPrep 20.0.2, MATLAB R2018a, Statistical Parametric Mapping (SPM12) software package (https://www.fil.ion.ucl.ac.uk/spm/) and Python 3.7.9. The following open sources were used for analyses involving the HCP-MMP1 parcellation: https://neurovault.org/images/29489/, https://figshare.com/articles/dataset/HCP-MMP1_0_projected_on_MNI2009a_GM_volumetric_in_NIfTI_format/3501911/4. 3D Slicer 4.11 was used for visualization. Behavioral data were analyzed using MATLAB R2020b, CircStat Toolbox implemented in Matlab and BeatRoot 0.5.8 (https://code.soundsoftware.ac.uk/projects/beatroot). Statistical analysis was performed using R software version 4.0.4, GraphPad Prism version 9.4.0 for Windows and the Statistical Package for the Social Sciences (IBM SPSS statistics 20.0).

For manuscripts utilizing custom algorithms or software that are central to the research but not yet described in published literature, software must be made available to editors and reviewers. We strongly encourage code deposition in a community repository (e.g. GitHub). See the Nature Portfolio guidelines for submitting code & software for further information.

Data

Policy information about availability of data

All manuscripts must include a data availability statement. This statement should provide the following information, where applicable:

- Accession codes, unique identifiers, or web links for publicly available datasets
- A description of any restrictions on data availability
- For clinical datasets or third party data, please ensure that the statement adheres to our policy

Musical stimuli, statistical parametric maps and the Python script used to generate Figure 2 are available to download from the Open Science Framework (OSF): https://osf.io/qnbwv/?view_only=e67e4737c321450f8488a80dcbfd5f5d. Behavioral and imaging raw data that support the findings of this study are available from the corresponding author upon reasonable request.

Field-specific reporting

Please select the one below that is the best fit for your research. If you are not sure, read the appropriate sections before making your selection.

☒ Life sciences ☐ Behavioural & social sciences ☐ Ecological, evolutionary & environmental sciences

For a reference copy of the document with all sections, see nature.com/documents/nr-reporting-summary-flat.pdf

Life sciences study design

All studies must disclose on these points even when the disclosure is negative.

Sample size	The overall sample size (N=71) was determined based on literature recommendations (Turner et al., 2018, Durnez et al., 2016) and prior lab experience with similar study designs. For the multiple linear regression analysis (combined behavioral and neuroimaging study), power analysis (power 0.80, alpha 0.05) indicated that the available sample size (53-58 subjects) is expected to detect medium-size effects.
Data exclusions	Participants exhibiting head motion of >2 mm were excluded from analysis of the relevant task (four, five and six participants in the rhythm melody and harmony paradigms, respectively).
Replication	No replication of experiments was performed. In the combined behavioral and fMRI study, to validate the regression results, a repeated region of interest analysis was performed based directly on MNI coordinates to avoid the projection of the surface-based HCP-MMP1 parcellation onto the volumetric Montreal Neurological Institute (MNI) space.
Randomization	Subject randomization was irrelevant for this single group study. The order of stimuli presentation in all paradigms was either randomized or pseudorandomized.
Blinding	Blinding was not relevant for the current study, as we were interested in whole group effects.

Reporting for specific materials, systems and methods

We require information from authors about some types of materials, experimental systems and methods used in many studies. Here, indicate whether each material, system or method listed is relevant to your study. If you are not sure if a list item applies to your research, read the appropriate section before selecting a response.

Materials & experimental systems

n/a	Involved in the study	
☒	☐	Antibodies
☒	☐	Eukaryotic cell lines
☒	☐	Palaeontology and archaeology
☒	☐	Animals and other organisms
☐	☒	Human research participants
☒	☐	Clinical data
☒	☐	Dual use research of concern

Methods

n/a	Involved in the study	
☒	☐	ChIP-seq
☒	☐	Flow cytometry
☐	☒	MRI-based neuroimaging

Human research participants

Policy information about studies involving human research participants

Population characteristics	A total of 71 right-handed healthy volunteers (median age, 25.0 years (range, 18-44); female, 41; median general education, 14 years (range, 12-18 years), median musical education, 2.0 years (range, 0-12 years)) participated in the study. All subjects had normal or corrected-to normal vision, reported normal hearing, had no history of neurological or psychiatric disorder, no

history of substance/alcohol abuse and no structural brain abnormality. All subjects were eligible for MRI scanning and did not use medications that may interfere with the study. Applicants with professional background in music or dance were excluded.

Recruitment	Participants were recruited via local and online advertising. As frequently encountered in neuroimaging studies, most of our participants are young adults, many of them are university students. A self-selection bias cannot be ruled out, particularly a bias related to special interest in music, however, screening of music perception skills revealed similar performance to previously reported non-musicians (Law and Zentner, 2012, Kunert et al., 2016). Based on previous literature, we do not expect musical education, general education or age to significantly affect the current study's results.
Ethics oversight	The study was approved by the Tel Aviv Sourasky Medical Center institutional review board (0017-18- TLV) and all participants provided written informed consent.

Note that full information on the approval of the study protocol must also be provided in the manuscript.

Magnetic resonance imaging

Experimental design

Design type	Task-fMRI. Block-design paradigms.
Design specifications	Rhythm paradigm:<br>Four conditions (4 levels of rhythmic complexity), four blocks per condition, block duration 18sec, inter-block interval 9sec.<br>Melody paradigm:<br>Four conditions (4 levels of melodic complexity), four blocks per condition, block duration 15sec, inter-block interval 9sec.<br>Harmony paradigm:<br>Four conditions (4 levels of harmonic complexity), four blocks per condition, block duration 18sec, inter-block interval 9sec.
Behavioral performance measures	Participants were not required to perform any task during scanning.

Acquisition

Imaging type(s)	Functional and structural.
Field strength	3T
Sequence & imaging parameters	Magnetization Prepared RApid Gradient Echo (MP-RAGE) sequence: TR/TE = 1860/2.74 ms, flip angle = 8°, FOV = 256 × 256 mm, voxel size = 1x1x1 mm, 176 slices.<br>T2*-weighted Gradient Echo Echo Planar Imaging (EPI) sequence: TR/TE = 3000/35 ms, flip angle = 90°, 96 × 96 matrix, FOV = 220 × 220 mm, 46 slices of 3 mm thickness, interleaved ascending order, no gap, whole-brain coverage, parallel imaging factor (iPAT) of 2.<br>Gradient Echo Field Mapping: TR = 400 ms, TE1 = 4.92 ms, TE2 = 7.38 ms, flip angle = 60°, 64 × 64 matrix, FOV = 220 × 220 mm, 36 slices of 3 mm thickness.
Area of acquisition	Whole-brain scan

Diffusion MRI ☐ Used ☒ Not used

Preprocessing

Preprocessing software	Preprocessing of BIDS-converted MRI data was performed using fMRIPrep (Esteban et al., 2019). fMRIPrep uses a combination of tools from several neuroimaging software packages, including FSL, ANTs, Freesurfer, Nilearn and AFNI. The structural T1-weighted (T1w) image was corrected for intensity non-uniformity (INU) with N4BiasFieldCorrection (ANTs 2.2.0), and used as T1w-reference throughout the workflow. The T1w-reference was then skull-stripped with a Nipype implementation of the antsBrainExtraction.sh workflow, using OASIS30ANTs as target template. Brain tissue segmentation of cerebrospinal fluid (CSF), white-matter (WM) and gray-matter (GM) was performed on the brain-extracted T1w using fast (FSL 5.0.9). Spatial normalization to a standard space was performed as detailed below. For functional data preprocessing, first, a reference volume and its skull-stripped version were generated using a custom methodology of fMRIPrep. A B0-nonuniformity map (fieldmap) was estimated based on a phase-difference map calculated with a dual-echo gradient-recall echo sequence, processed with a custom workflow of SDCFlows inspired by the epidewarp.fsl script and further improvements in HCP Pipelines. Based on the estimated susceptibility distortion, a corrected BOLD reference was calculated for a more accurate co-registration with the anatomical reference. The BOLD reference was then co-registered to the T1w reference using flirt (FSL 5.0.9) with the boundary-based registration cost-function. Co-registration was configured with nine degrees of freedom to account for distortions remaining in the BOLD reference. Head-motion parameters with respect to the BOLD reference (transformation matrices, and six corresponding rotation and translation parameters) were estimated before any spatiotemporal filtering using mcflirt (FSL 5.0.9). BOLD runs were slice-time corrected using 3dTshift from AFNI 20160207. The BOLD time-series were resampled onto their original, native space by applying a single, composite transform to correct for head-motion and susceptibility distortions and then resampled into the standard space. Spatial smoothing (6 mm full width half-maximum Gaussian kernel) was performed using SPM12.

Normalization	Volume-based spatial normalization to the MNI space was performed through nonlinear registration with antsRegistration (ANTs 2.2.0) using brain-extracted images.
Normalization template	ICBM 152 Nonlinear Asymmetrical template version 2009c.
Noise and artifact removal	We included the following nuisance regressors estimated during preprocessing with fMRIPrep in the first-level analysis: time series derived from the whole brain, WM and CSF masks, six rigid-body motion parameters, the first temporal derivatives and quadratic terms for each of the above, framewise displacement (FD) and the standardized derivative of root mean square variance over voxels (std-DVARS).
Volume censoring	Volume censoring was based on framewise displacement (FD) values, such that frames that exceeded a threshold of 0.9 mm FD were annotated as motion outliers.

Statistical modeling & inference

Model type and settings	Univariate analysis. First and second level analyses were conducted using SPM12. The BOLD fMRI signal was modeled with a general linear model (GLM), using a canonical hemodynamic response function and a standard temporal filter of 128 s. Nuisance motion regressors were included in each first-level model as detailed above. Individual statistical parametric maps were calculated for the contrast of all conditions vs. baseline. Second level analysis used mixed effect modelling across subjects.
Effect(s) tested	The rhythm, melody and harmony paradigms were analyzed here to unveil common neural correlates of music perception. In order to achieve maximal activations we used the contrast of all conditions together vs. baseline.

Specify type of analysis: ☐ Whole brain ☐ ROI-based ☒ Both

Anatomical location(s)	Anatomical masks of the regions of interest were drawn from the HCP-MMP1 parcellation.
Statistic type for inference (See Eklund et al. 2016)	Voxel-wise inference
Correction	Cluster level correction, family-wise error (FWE), p < 0.05.

Models & analysis

n/a	Involved in the study
☒	☐ Functional and/or effective connectivity
☒	☐ Graph analysis
☒	☐ Multivariate modeling or predictive analysis
